# Transcriptome analysis reveals the activation of neuroendocrine-immune system in shrimp hemocytes at the early stage of WSSV infection

**DOI:** 10.1186/s12864-019-5614-4

**Published:** 2019-03-28

**Authors:** Fuxuan Wang, Shihao Li, Jianhai Xiang, Fuhua Li

**Affiliations:** 1 Key Laboratory of Experimental Marine Biology, Institute of Oceanology, Chinese Academy of Sciences, Qingdao, 266071 China; 2 Laboratory for Marine Biology and Biotechnology, Qingdao National Laboratory for Marine Science and Technology, Qingdao, 266237 China; 3 University of Chinese Academy of Sciences, Beijing, 100049 China; 4 Center for Ocean Mega-Science, Chinese Academy of Sciences, Qingdao, 266071 China

**Keywords:** *Litopenaeus vannamei*, Hemocytes, WSSV, Neuroendocrine-immune system

## Abstract

**Background:**

Functional communications between nervous, endocrine and immune systems are well established in both vertebrates and invertebrates. Circulating hemocytes act as fundamental players in this crosstalk, whose functions are conserved during the evolution of the main groups of metazoans. However, the roles of the neuroendocrine-immune (NEI) system in shrimp hemocytes during pathogen infection remain largely unknown.

**Results:**

In this study, we sequenced six cDNA libraries prepared with hemocytes from *Litopenaeus vannamei* which were injected by WSSV (white spot syndrome virus) or PBS for 6 h using Illumina Hiseq 4000 platform. As a result, 3444 differentially expressed genes (DEGs), including 3240 up-regulated genes and 204 down-regulated genes, were identified from hemocytes after WSSV infection. Among these genes, 349 DEGs were correlated with innate immunity and categorized into seven groups based on their predictive function. Interestingly, 18 genes encoded putative neuropeptide precursors were induced significantly by WSSV infection. Furthermore, some genes were mapped to several typical processes in the NEI system, including proteolytic processing of prohormones, amino acid neurotransmitter pathways, biogenic amine biosynthesis and acetylcholine signaling pathway.

**Conclusions:**

The data suggested that WSSV infection triggers the activation of NEI in shrimp, which throws a light on the pivotal roles of NEI system mediated by hemocytes in shrimp antiviral immunity.

**Electronic supplementary material:**

The online version of this article (10.1186/s12864-019-5614-4) contains supplementary material, which is available to authorized users.

## Background

In the past few decades, numerous studies have focused on the invertebrate neuroendocrine-immune (NEI) biology. The NEI regulatory network consists of nervous system, endocrine system and immune system, which are integrated into a single system [1]. There is a reciprocal regulation in the regulatory network to maintain host homeostasis. The regulatory network is conserved in highly divergent and evolutionarily distant animals such as molluscs, crustaceans, insects and mammals [2]. The reciprocal regulation involves a large number of signal molecules, such as neurotransmitters, hormones and cytokines [3]. In vertebrates, nerve pathways, hormonal circuits, cytokines, neuropeptides and chemokines are mediators connecting various elements of NEI system [4]. Analogous NEI connections have also be found in invertebrate phyla [5]. For instance, multiple neurotransmitters are released from nervous and endocrine tissues and conduct neural immune regulation through a nervous-hemocyte neuroendocrine immunomodulatory axis (NIA)-like pathway in mollusks [6].

Pacific white shrimp (*Litopenaeus vannamei*) belongs to the Penaeidae family of decapod crustaceans and has become the most commonly cultured shrimp species in the world. However, shrimp diseases caused by viruses, bacteria, fungi and protozoa, have occurred frequently in the past 20 years, which hinders the development of the global shrimp industry [7]. White spot syndrome virus (WSSV; genus *Whispovirus*, family *Nimaviridae*) is one of the destructive pathogens causing 100% mortalities within 3–10 days [8] and substantial economic losses of billions of US dollars [9]. Understanding the host immune responses to pathogen infection is the foundation to develop a strategy for disease control and prevention. Next-generation sequencing technology has been widely applied to understand the molecular responses in shrimp against pathogens, such as WSSV [10], Taura syndrome virus (TSV) [11], *Vibrio parahaemolyticus* (*V.p*) [12]. Considerable transcripts involved in immune defense have been identified in specific tissues of disease-stressed shrimp. However, more efforts are still needed to understand the defense mechanisms of shrimp during pathogen infection.

Evidence shows that invertebrate hemocytes play a crucial role both in cellular and humoral immunity [13, 14]. The cellular immune responses include apoptosis, encapsulation, phagocytosis and nodule formation, while the humoral responses mediated by hemocytes consist of the prophenoloxidase (proPO) system, the clotting cascade and secretion of antimicrobial peptides [15, 16]. Hemocytes also function as neuroendocrine system. For example, the release of norepinephrine (NE) from hemocytes to hemolymph was found and the key enzyme dopamine β-hydroxylase (DBH) for NE synthesis was identified in hemocytes [17, 18]. Overall, circulating hemocytes hailed as “mobile immune-brain” are fundamental players in NEI network in invertebrates [6]. However, knowledge about the responses of NEI system in shrimp hemocytes during WSSV infection is still limited. In this study, we examined the transcriptional profiles of the hemocytes in *L. vannamei* during WSSV infection, with aims to identify the molecular components of NEI network in shrimp hemocytes and explore its potential roles during the early stage of WSSV infection. The data will not only increase our understanding on the molecular mechanisms of the immune responses in shrimp hemocytes to WSSV infection, but also be useful for developing anti-WSSV approaches.

## Results and discussion

### RNA-Seq and de novo assembly

The detail information of sequencing and assembly of the transcriptome from hemocytes of *L. vannamei* was shown in Table 1. Using Illumina HiSeq™ 4000, a total of 304,011,446 raw reads were obtained from the Pacific white shrimp, of which 137,558,608 reads were from PBS-challenged hemocytes (PHc group) and 166,452,838 reads were from WSSV-challenged hemocytes (WHc group). After cleaning of these inappropriate reads, the percentage retained of reads from PHc and WHc group was 97.10 and 96.91%, respectively. A total of 44,793 unigenes were assembled, with half of the total assembly length (N50) of 2406 bp and an average length of 1273 bp. The distribution of predicted coding sequence (CDS) lengths was shown in Additional file 1.

**Table 1:** Summary of sequencing and assembly of the transcriptome from *L. vannamei*

	Raw Reads	Clean Reads	Percentage retained	Gene number	Ratio
PHc-1	47,181,514	45,798,586	97.07%	25,525	56.98%
PHc-2	41,051,168	39,912,832	97.23%	24,555	54.82%
PHc-3	49,325,926	47,856,124	97.02%	31,895	71.21%
PHc	137,558,608	133,567,542	97.10%	35,596	79.47%
WHc-1	59,987,346	58,029,050	96.74%	37,885	84.58%
WHc-2	56,726,760	55,039,876	97.03%	33,889	75.66%
WHc-3	49,738,732	48,241,248	96.99%	33,763	75.38%
WHc	166,452,838	161,310,174	96.91%	42,851	95.66%

Note: Ratio = gene number expressed in each sample/all reference gene number (44,793)

### Functional annotation of all unigenes

For annotation, all unigenes were searched using the BLAST algorithm against Nr, Swiss-Prot, KOG, and KEGG databases. The annotation results were shown through the Venn diagram (Additional file 2). Out of 44,793 unigenes, 14,741 unigenes (32.91%) were annotated in at least one database, and 6505 unigenes (14.52%) were annotated in all databases. However, there were still 30,052 unigenes (67.09%) that were not annotated based on similarity search, which implied that these unigenes might be helpful in the discovery of *L. vannamei*-specific genes.

### Identification and functional annotation of DEGs

To identify DEGs involved in WSSV infection in shrimp, we used RPKM value for comparing the expression levels between PHc group and WHc group. A total of 3444 DEGs were obtained, including 3240 differentially up-regulated genes (DUGs) and 204 differentially down-regulated genes (DDGs) as shown in Additional file 3. The data indicated that shrimp hemocytes mainly exhibited positive responses to WSSV infection at 6 h post injection (hpi).

To verify the accuracy of transcriptome data, 13 DUGs and 2 DDGs were selected randomly from 3240 DEGs for SQ-PCR. The results of SQ-PCR are shown in Additional file 4. All DEGs showed consistent expression patterns with transcriptome data, confirming that our results were valid.

To know the function of DEGs during the early stage of WSSV infection in shrimp, we performed GO enrichment analysis using Blast2GO. A total of 1273 GO terms belonging to three main GO categories (cellular component 137; molecular function 297; biological process 839) were obtained in response to WSSV infection (Additional file 5) and the top five most significantly enriched GO terms with *p*-value < 0.05 were listed in Table 2. For example, “cholinesterase activity” in the GO molecular function terms is essential for the metabolism of the neurotransmitter acetylcholine, suggesting that the neuroendocrine system might play an important role in the early stage of WSSV infection. At the same time, we also carried out GO term classification statistics based on up- and down-regulated genes. As shown in Additional file 6 the functional distribution of all DEGs including DUGs and DDGs was similar. Of the GO biological process related genes, most were involved in “cellular process” and “metabolic process”. Most of the cellular component related genes were associated with “cell”, “cell part” and “organelle”. And “binding” and “catalytic activity” in the molecular function ontology were the major enriched terms.

**Table 2:** Top 5 significantly enriched GO terms in WSSV-challenged hemocytes

	GO-Cellular Component	GO-Molecular Function	GO-Biological Process
1	mitochondrial part	carboxylic ester hydrolase activity	cellular component biogenesis
2	nucleus	cholinesterase activity	RNA metabolic process
3	mitochondrion	transcription factor activity, protein binding	ribonucleoprotein complex biogenesis
4	intracellular organelle part	aminoacyl-tRNA ligase activity	cellular component organization or biogenesis
5	cell-cell junction	ligase activity, forming carbon-oxygen bonds	RNA processing

To understand the potential gene interactions during WSSV infection in shrimp, we conducted the KEGG pathway enrichment analysis using KASS tool. The DEGs were mapped to 143 different pathways (Additional file 7) and the top 20 pathways were shown in Additional file 8. Many pathways were closely associated to metabolism, including lipid metabolism (“Arachidonic acid metabolism”, “Steroid biosynthesis”), amino acid metabolism (“Arginine and proline metabolism”, “Alanine, aspartate and glutamate metabolism” and “Taurine and hypotaurine metabolism”) and nucleotide metabolism (“Pyrimidine metabolism”), except for the first four pathways related to protein synthesis. Our results revealed that WSSV might affect several basic cellular metabolic processes during the early stage of infection to fulfill its successful replication, which have been reported by previous studies as well [19–21].

To further understand the immune response of shrimp against WSSV challenge, we summarized immune-related DEGs by category. These selected DEGs were categorized into seven groups, mainly including pattern recognition, related to signal transduction, antimicrobial peptides, ubiquitin mediated proteolysis, related to phagocytosis and proteases/protease inhibitors (Additional file 9). It is well known that the activation of the innate immune response depends on the recognition of pathogens by the pattern recognition receptors (PRRs) [22]. In invertebrates, several groups of PRRs have been identified, including peptidoglycan recognition proteins (PGRPs), Gram-negative binding proteins (GNBP) or lipopolysaccharide and β-1,3-glucan binding proteins (LGBPs), C-type lectins, galectins, thioester containing proteins (TEPs), fibrinogen-related proteins (FREPs), scavenger receptors (SRs), Down syndrome cell adhesion molecules (DSCAMs) and Toll like receptors (TLRs) [22]. In our data, the members of the six above-mentioned PRR families have been identified in shrimp, i.e., LGBPs, C-type lectins, FREPs, SR, DSCAM and TLR, and most of these identified PRRs showed up-regulation at 6 h after WSSV infection (Additional file 9), indicating their important roles in response to WSSV infection. Phagocytosis is a major way used to remove pathogens and cell debris in both vertebrates and invertebrates [23]. In shrimp, hemocytes are the main performers of phagocytosis and tend to protect against various pathogens [24]. Here, 90 DEGs related to phagocytosis were listed in Additional file 9 suggesting stimulation of hemocytes by WSSV infection probably increases their phagocytic processes. Therefore, we concluded that the immune system of shrimp is activated by WSSV.

### The activation of NEI system in shrimp hemocytes triggered by WSSV infection

After analysis of all DEGs from shrimp hemocytes, we discovered many biological processes that are closely related to the NEI system. These processes include proteolytic processing of prohormones, amino acid neurotransmitter pathways, biogenic amine biosynthesis and acetylcholine signaling pathway, all of which are affected by viral infection and are shown in proposed diagrams (Figs. 2–7).

#### Identification of neuropeptide precursors and their proteolytic processing in *L. vannamei*

**Fig. 1:**
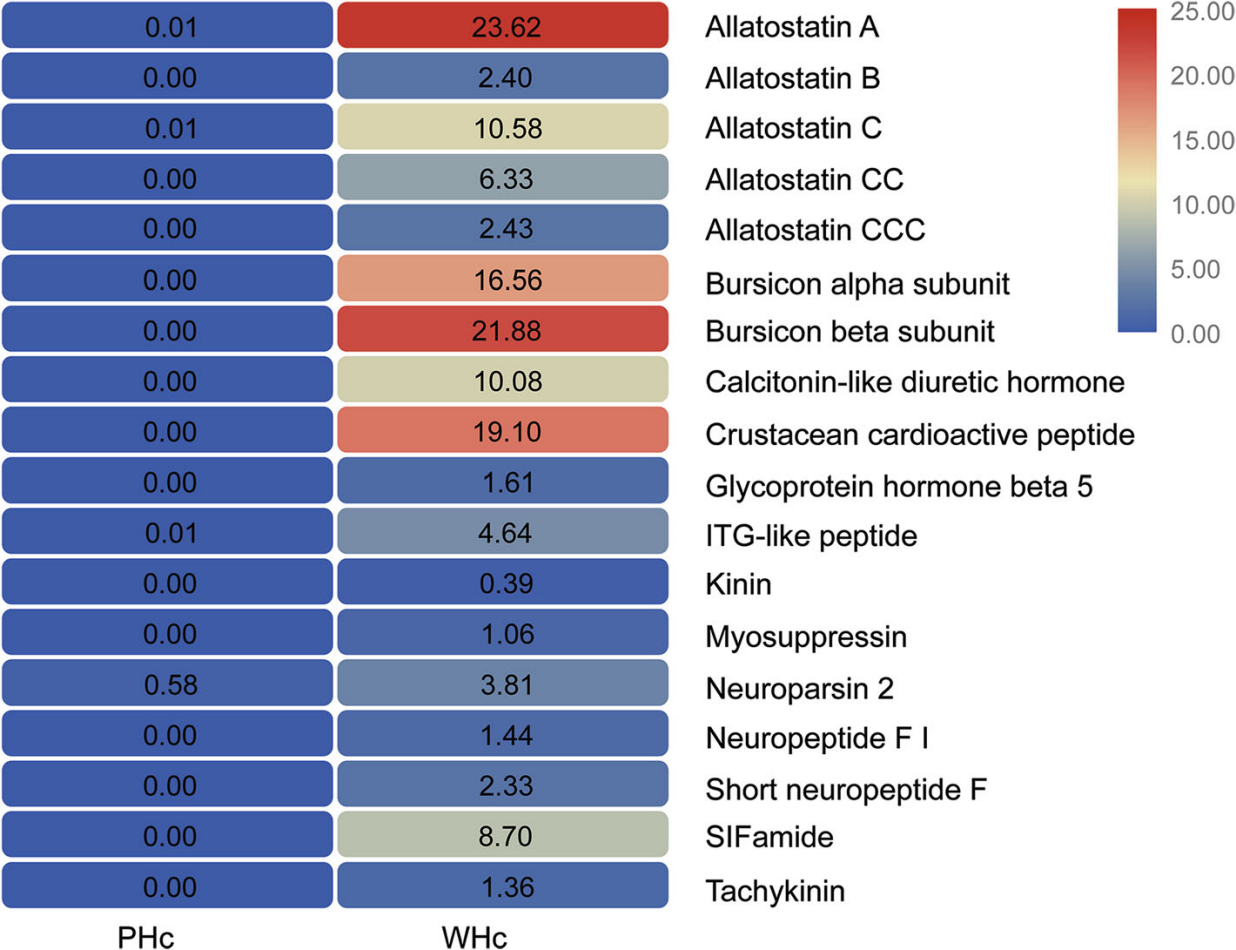
Heatmap showing fold changes of neuropeptide precursors. The values in the figure indicates RPKM

The hypothalamus-pituitary-adrenal axis constitutes the most powerful circuit regulating the immune system and the neuropeptides in this axis are potent and direct immunoregulators [25, 26]. In total, 18 transcripts encoding neuropeptide precursors, including 16 complete sequences and two partial sequences, were identified (Table 3, Additional file 10), with a large number of distinct mature peptides predicted. Most neuropeptide precursors were not expressed in unstimulated hemocytes and few were expressed at very low level, while the mRNA abundance of all identified neuropeptide precursors increased significantly at 6 h after viral infection (Fig. 1). Shrimp suffering from WSSV infection display various clinical signs, especially plenty of obvious white spots or patches, a reduction in feed uptake, and thinning and delayed clotting of hemolymph [9]. Recently, an in vivo Warburg-like effect that was induced in shrimp hemocytes by WSSV via the PI3K-Akt-mTOR pathway was reported, and the effect characterized by a series of metabolic changes was essential for virus because it can provide enough energy and materials for successful viral replication [19]. Among these identified neuropeptides, most of them were associated with the regulation of feeding, including allatostatins, calcitonin-like diuretic hormone, kinin, myosuppressin, neuropeptide F I, short neuropeptide F, SIFamide, tachykinin [27]. Specially, both allatostatin A and myosuppressin were able to perform a function of reducing feeding by inhibiting gut mobility [28, 29]. Besides, allatostatin A is a modulator of AKH and DILP signaling [30] and may contribute to metabolic changes induced by WSSV. It should be noted that all five complete allatostatin precursors, namely allatostatin-A, allatostatin-B, and allatostatin-C, CC, CCC were discovered simultaneously at the first time in crustaceans and their expression profiles were consistent (Fig. 1). Allatostatins are a group of three structurally distinct peptide families originally identified as inhibitors of juvenile hormone (JH) synthesis in specific insect orders [31]. Mounting evidence suggests that allatostatins are pleiotropic and can orchestrate diverse physiological, neuronal or behavioural processes. For instance, allatostatin A had been implicated in the regulation of metabolism, sleep and feeding behaviors [28, 30], whereas allatostatin C, an immunosuppressive substance released by nociceptors or hemocytes, modulated nociception and immunity in *Drosophila* [32]. Our findings provided a new understanding of the allatostatin family’s responses to viral infections. Overall, these neuropeptides, produced in the hemocytes infected by WSSV, were released into the hemolymph to act on various target tissues expressed the corresponding receptors, which lead to pathological features and eventually death. These data suggested that neuropeptides encoded precursors are likely to be regulated by WSSV, which in turn facilitate viral replication.

**Table 3:** Putative neuropeptide precursors induced by WSSV in the hemocytes transcriptome of *L. vannamei*

Gene ID	Description	Size(bp)/ (aa)	Species	*E*-value	Ident	Accession
Unigene0020648	Allatostatin A	3570/620	*Panulirus interruptus*	9e-176	52%	BAF64528.1
Unigene0002365	Allatostatin B	1347/332	*Scylla paramamosain*	6e-89	54%	ALQ28584.1
Unigene0022818	Allatostatin C	1158/139	*Scylla paramamosain*	2e-29	47%	ALQ28578.1
Unigene0028974	Allatostatin CC	1387/73	*Cherax quadricarinatus*	1e-28	75%	AWK57504.1
Unigene0018310	Allatostatin CCC	773/105	*Cherax quadricarinatus*	1e-56	79%	AWK57503.1
Unigene0025940	Bursicon-α	1211/142	*Penaeus monodon*	5e-95	96%	AKJ74864.1
Unigene0026844	Bursicon-β	1119/145	*Penaeus monodon*	5e-100	99%	ALO17552.1
Unigene0057574	Calcitonin-like diuretic hormone	1235/154	*Homarus americanus*	3e-63	91%	CX46386.1
Unigene0039878	Crustacean cardioactive peptide	1195/139	*Penaeus monodon*	2e-97	100%	ALP06206.1
Unigene0049442	Glycoprotein hormone β5	436/80*	*Penaeus monodon*	1e-46	100%	ALO17559.1
Unigene0032916	ITG-like peptide	2715/199	*Cryptotermes secundus*	3e-56	52%	XP_023703406.1
Unigene0004333	Kinin	1224/147*	*Scylla paramamosain*	1e-32	61%	ALQ28594.1
Unigene0029204	Myosuppressin	796/99	*Homarus americanus*	6e-39	68%	ACX46385.1
Unigene0024101	Neuroparsin 2	850/99	*Cherax quadricarinatus*	7e-32	58%	AWK57531.1
Unigene0057187	Neuropeptide F I	1183/90	*Penaeus vannamei*	6e-58	100%	AEC12204.1
Unigene0056553	Short neuropeptide F	596/129	*Cherax quadricarinatus*	2e-54	66%	AWK57543.1
Unigene0020284	SIFamide	1619/76	*Procambarus clarkii*	4e-30	80%	Q867W1.1
Unigene0024954	Tachykinin A	1251/181	*Panulirus interruptus*	1e-74	56%	BAD06362.1

Note: incomplete precursors are marked by asterisk (*)

**Fig. 2:**
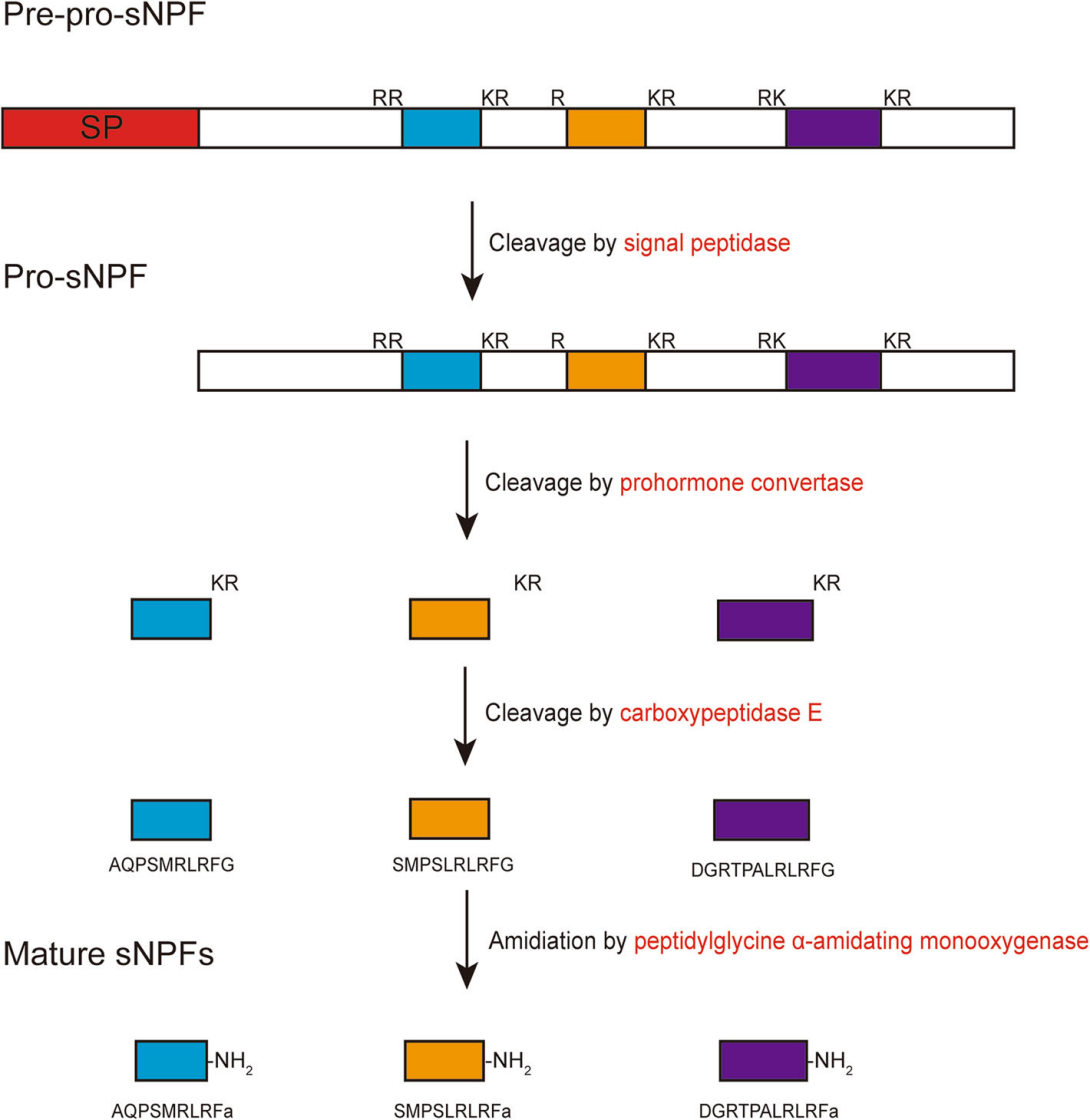
Post-translational processing steps of sNPF precursor in shrimp. Representative sNPF precursor consists of eight sections, including a signal peptide, three mature sNPF copies and four linkers. Red font denotes DUGs

Neuropeptides are derived from pre-pro-peptides, which are large, inactive precursor molecules that must be proteolytically cleaved and post-translationally modified to yield the mature peptides [33]. The neuropeptide processing enzymes were also induced in shrimp hemocytes at the early stage of WSSV infection (Additional file 10). The processing of neuropeptide precursors appears to have been well conserved throughout the evolution as shown in Fig. 2 and previous studies [34]. The first event in the processing is the removal of the signal peptide by signal peptidase, resulting in the conversation of pre-pro-peptide to pro-peptide. A probable signal peptidase complex subunit 2, belonging to SPC25 superfamily, was induced by WSSV infection (Unigene0025582 in Additional file 10). Prohormone convertases (PCs) are thought to be responsible for cleavage of many pro-peptides at consensus paired basic residues or occasionally at monobasic sites, such as PC1, PC2, furin, PC4, PC5, paired basic amino acid cleaving enzyme 4 (PACE4) and PC7 in mammals [33]. They are also subject to various post-translational modifications before attaining the catalytic or binding ability [35]. In our study, two precursors of these enzymes (PC1 and PC2) and a neuroendocrine protein (7B2) were identified in hemocytes as DUGs (Unigene0010022, Unigene0036815 and Unigene0029859, respectively, in Additional file 10). PC2 precursor is transported as a complex with its binding protein 7B2 to acidic immature secretory granules for activation, which is different from other PCs [36]. PC1 and PC2 are mostly localized within immature and dense-core secretory granules of neural and endocrine cells in mammals [35], providing direct molecular evidence for the versatility of shrimp hemocytes. The expression profiles of PCs identified in virus-infected hemocytes indicated that they might participate in WSSV early infection in shrimp hemocytes via processing host neuropeptide precursors. In addition, these PCs might also cleave WSSV envelope proteins, because the intact viral proteins are incapable of accomplishing these processes, including the exposure of their membrane-penetrating peptide region and escape into the cytoplasm of host cells [37]. Subsequently, carboxypeptidase E only cleaves carboxy-terminal basic amino acids from peptide intermediates processed by PCs [38]. At last, an impressive range of different types of posttranslational modification are required for full biological activity of neuropeptides, including glycosylation, sulfation, phosphorylation, cyclization, amidation and acylation [33]. Amidation is a critical, late-stage post-translational modification for many neuropeptides, which is catalyzed by the sequential action of two enzymes, peptidylglycine-α-hydroxylating monooxygenase (PHM) and peptidyl-α-hydroxyglycine α-amidating lyase (PAL), which are derived from a single bifunctional precursor [39]. Here we identified a shrimp PAL2 gene as a DUG (Unigene0023504 in Additional file 10) in hemocytes after WSSV infection suggesting a crucial role of shrimp PAL2 mediated amidation of bioactive peptides in the early stage of WSSV infection.

#### Analysis of amino acid neurotransmitters and their pathways in *L. vannamei*

##### Glutamate metabolism

**Fig. 3:**
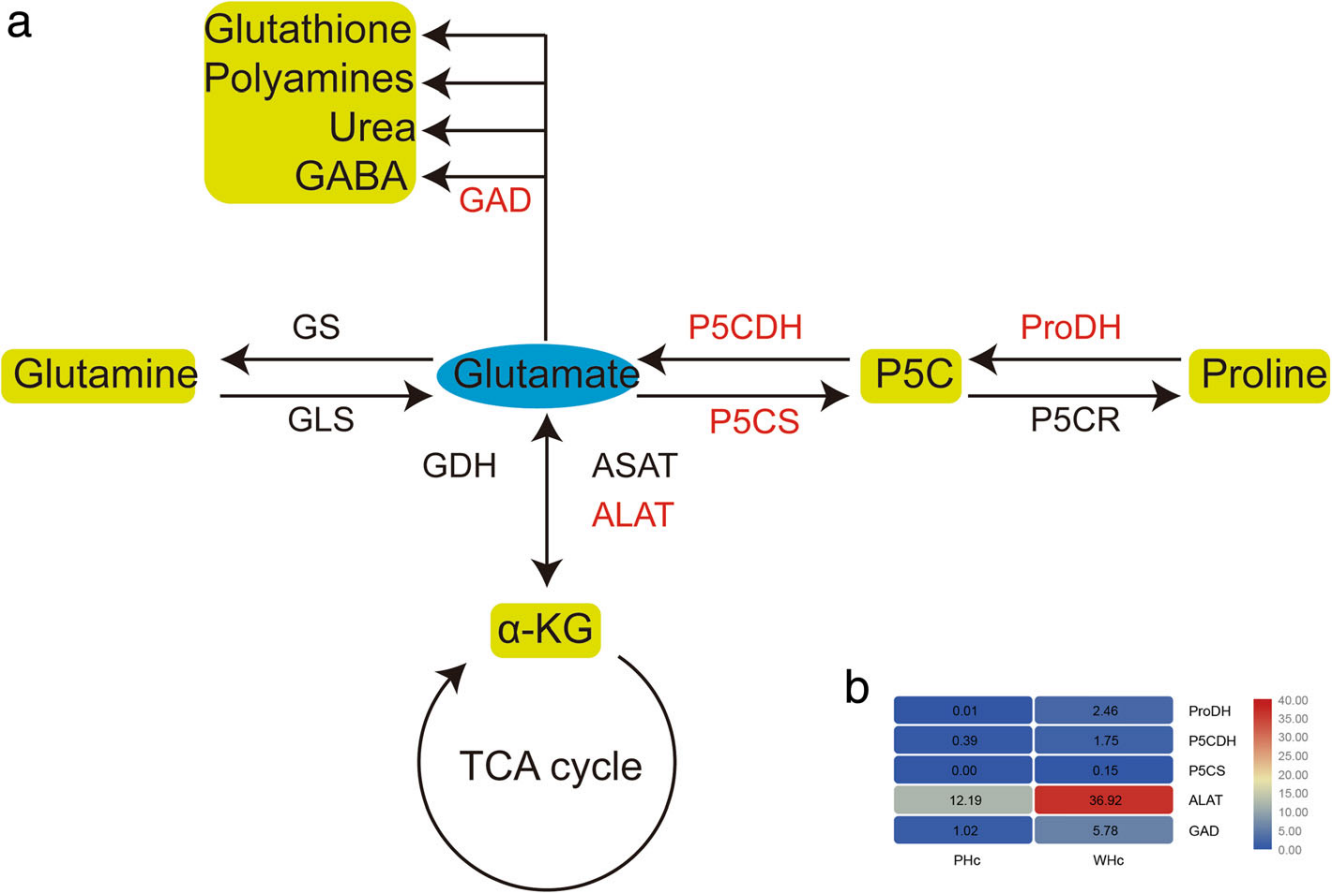
The metabolic pathway of amino acid glutamate (**a**) and expression changes of related genes (**b**). The mRNA levels of genes shown in red were increased. The values in the figure indicates RPKM. GLS, glutaminase; GS, glutamine synthetase; P5CR, △^1^-pyrroline-5-carboxylate reductase

Several amino acids have been implicated as neurotransmitters in the mammalian central nervous system (CNS), which are of two categories: excitatory neurotransmitters (glutamate, aspartate) and inhibitory neurotransmitters (γ-aminobutyric acid, glycine) [40]. Glutamate is at the crossroad between multiple metabolic pathways [41] and critically involved in mechanisms of synaptic plasticity, memory, and neuronal or glial cell death [42]. Based on our transcriptome data and previous research [43], we drew a brief pathway map of glutamate metabolism (Fig. 3a). Three of the four enzymes involved in proline and glutamate catabolism, including proline dehydrogenase (ProDH), △^1^-pyrroline-5-carboxylate dehydrogenase (P5CDH) and △^1^-pyrroline-5-carboxylate synthetase (P5CS), were significantly up-regulated at 6 hpi (Fig. 3b). ProDH and P5CDH are two key mitochondrial enzymes in the cellular biogenesis of glutamate, and P5CS is the rate-limiting enzyme in proline biosynthesis [44]. Proline and glutamate could serve as important sources of substrate for the TCA cycle [45], suggesting their importance in energy metabolism. The data showed that the conversion between glutamate and proline was significantly accelerated by WSSV in shrimp hemocytes. Furthermore, we noted that alanine aminotransferase (ALAT), catalyzing the reversible transamination between alanine and α-ketoglutarate (α-KG) to form pyruvate and glutamate, was significantly up-regulated at 6 hpi (Fig. 3b). We speculate that the conversion of glutamate to α-KG mediated by ALAT seems to be an alternative pathway at 6 hpi that fuels the TCA cycle via α-KG and ultimately facilitates WSSV replication. Glutamate decarboxylase or glutamic acid decarboxylase (GAD) catalyzing in the conversion of glutamate to γ-aminobutyric acid (GABA) is described in more detail below.

##### Excitatory amino acids (EAAs) pathway

**Fig. 4:**
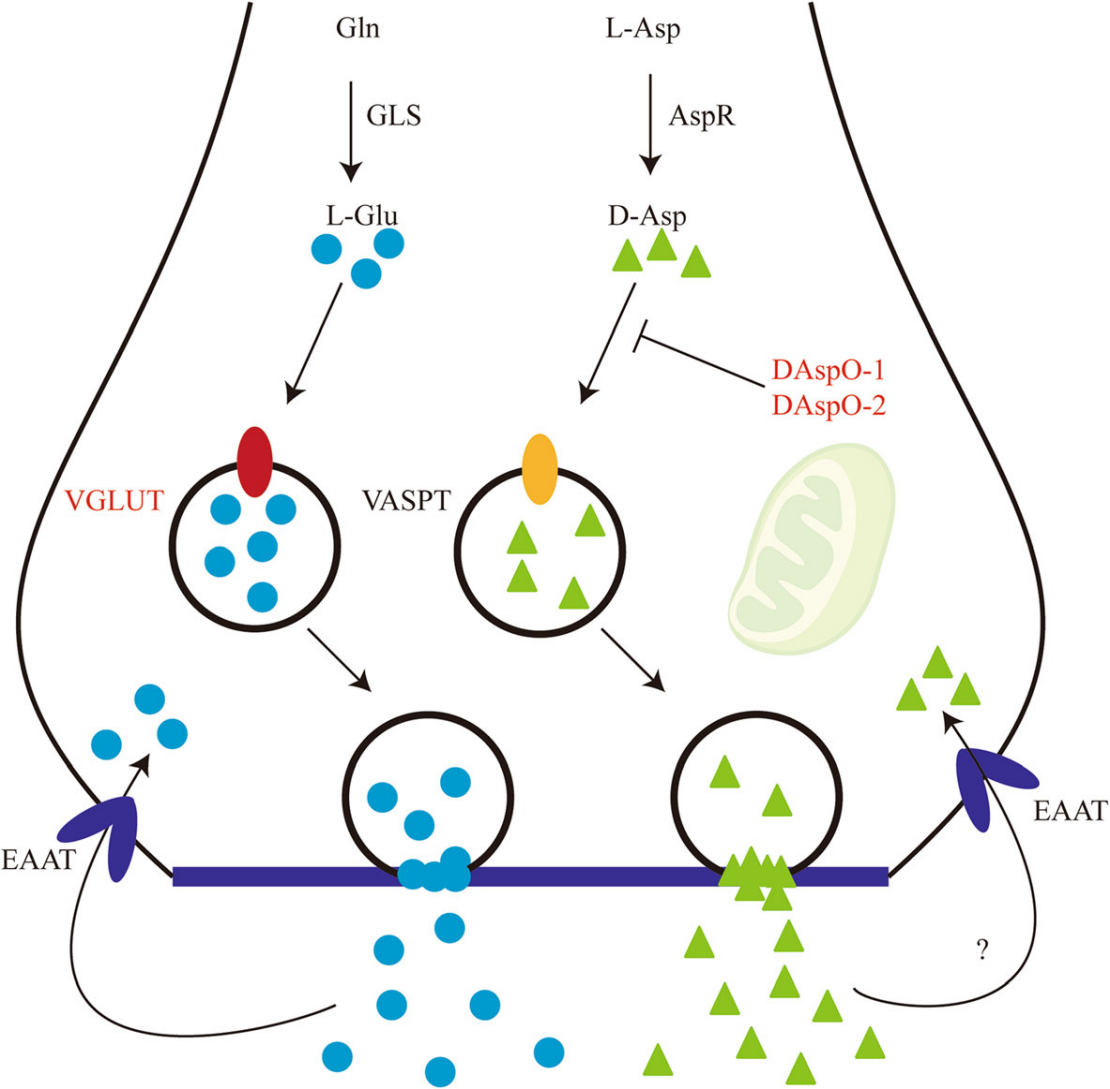
Schematic diagram of EAAs synthesis, degradation, release and transport at a presynaptic terminal. Red font denotes DUGs

Excitatory amino acid transmitters take up most fast synaptic transmission that occurs in the brain [46]. The excitatory amino acids (EAAs) pathway has been described in animals [47]. Glutaminase (GLS) converts glutamine (Gln) to glutamate (Glu) and aspartate racemase (AspR) catalyze D-Asp formation using L-Asp as a substrate, which are subsequently transported into synaptic vesicles for release by vesicular glutamate transporter (VGLUT) and vesicular aspartate transporter (VASPT), respectively [48]. Upon release, the neurotransmitters bind to corresponding receptors and are taken up by EAAT (excitatory amino acids transporter) into neurons where they can be recycled or metabolized via several enzymes. Among DUGs, one VGLUT (Unigene0001379), two ionotropic glutamate receptor subunits (Unigene0028013, Unigene0043289) and two D-aspartate oxidases (DAspOs, Unigene0012006, Unigene0026414) were identified in shrimp hemocytes (Fig. 4, Additional file 10). VGLUT is located on the cell membrane of synaptic vesicles and transports glutamate from the cytoplasm into synaptic vesicles [49]. Upon release from synaptic vesicles into the synaptic cleft by fusing with the plasma membrane of the presynaptic terminal, glutamate is probably bound to pre- and post-synaptic receptors [50]. Both excessive production and aberrant stimulation of glutamate receptor mediated by virus infection results in neuronal damage [51, 52]. Therefore, we propose that glutamatergic system might be used during WSSV infection. There is increasing evidence that D-Asp presents in the central nervous and reproductive systems of vertebrates and invertebrates [53]. The degradation of D-Asp is pivotal when determining if it is a neurotransmitter. D-Asp degradation is likely mediated by DAspO [53]. The distribution of DAspO is mainly in liver, kidney and brain of mammals, birds, fishes and amphibians [54]. However, three genes were present in shrimp hemocytes and two of which were induced obviously after WSSV infection. Consequently, aspartatergic system was likely to be negatively regulated in shrimp hemocytes by WSSV infection.

##### Inhibitory amino acids (IAAs) pathway

**Fig. 5:**
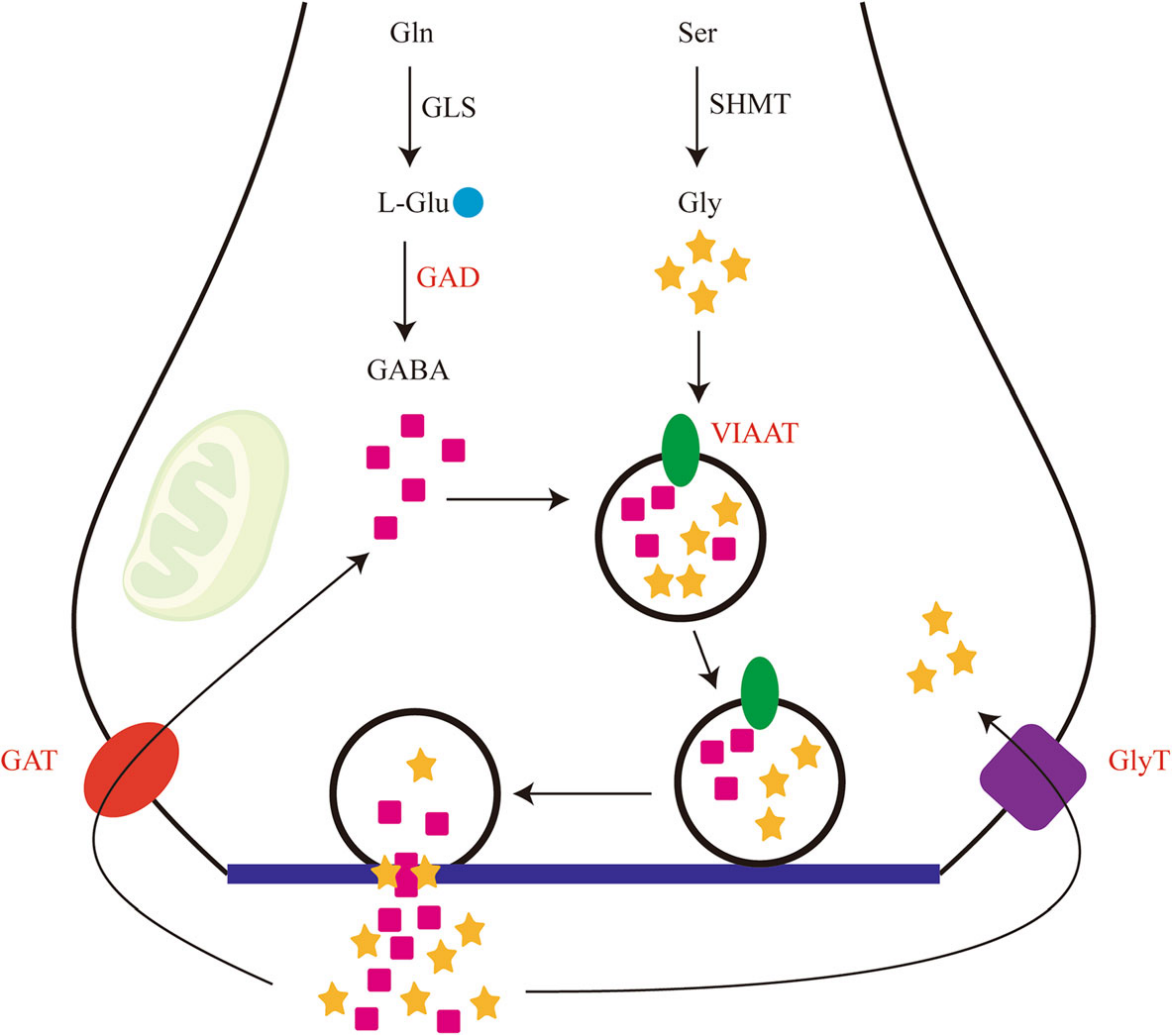
Schematic diagram of IAAs synthesis, release and transport at a presynaptic terminal. Red font denotes DUGs. SHMT, serine hydroxymethyl transferase

Inhibitory amino acid pathway consists of synthesis, release, reuptake, and metabolism of GABA and glycine, mediating chiefly inhibitory neurotransmission in the adult nervous system [47, 55]. In the present study, a pertinent metabolizing enzyme, three membrane-associated high affinity transporters and two receptor subunits in the IAA pathway were identified (Fig. 5, Additional file 10). GAD is an enzyme that converts the excitatory amino acid glutamate into GABA [47]. Two isoforms, GAD65 and GAD67, named for their molecular weights in kilodaltons, have been identified in vertebrates and function both in the nervous system and immunomodulation [56]. In invertebrates, there is only one GAD isoform identified and the GAD is always regarded as a useful molecular marker for GABAergic neurons [56]. The vesicular inhibitory amino acid transporter (VIAAT) or vesicular GABA transporter (VGAT) possesses H^+^-antiport activity, which ensures the active uptake of GABA and glycine into synaptic vesicles and their subsequent exocytotic release from the nerve terminals [57]. The glycine transporter GlyT mediates the reuptake of glycine into nerve terminals, which regulates its effective synaptic concentrations [58]. Like GlyT, the GABA transporter GAT is present in neurons and its activity is crucial to regulate the extracellular concentration of GABA under basal conditions and during ongoing synaptic events [59]. In GABAergic system, signalling of GABA via GABA type A receptor channels or G-protein-coupled type B receptors was implicated in multiple CNS functions [60]. These receptors were also expressed in the immune cells, such as T cells, dendritic cells (DCs), and exerted immunomodulatory function [60]. For instance, activation of GABA type A receptor regulated the migration of Toxoplasma-infected dendritic cells [61]. It is clear that the GABA signaling system is active in the immune cells and can affect a variety of functional properties of the cells like cytokine secretion, cell proliferation, phagocytic activity and chemotaxis [62, 63]. Accordingly, we assumed that WSSV infection might change several characteristics of shrimp hemocytes through GABAergic system. Glycine is critical in glycinergic system in causing membrane hyperpolarization in the mature nervous system [64]. Additionally, glycine is also a novel anti-inflammatory, immunomodulatory and cytoprotective nutrient. Evidence shows that glycine not only plays a role in regulating the production of cytokines by leucocytes, but also reduces inflammatory reactions and morbidity in pathogen-infected animals [65]. However, whether glycine can protect shrimp against viral infection requires further research.

#### Analysis of biosynthesis of biogenic amines in *L. vannamei*

**Fig. 6:**
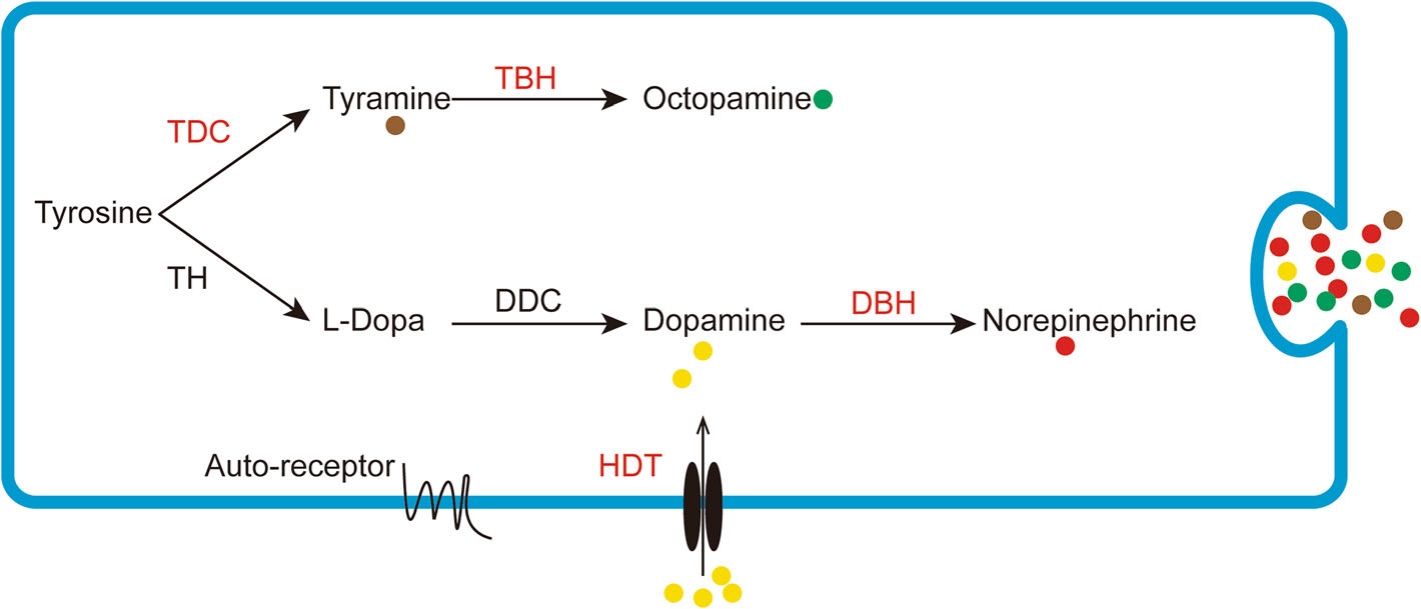
Biosynthesis of biogenic amines in shrimp. Red font denotes DUGs.TH, tyrosine hydroxylase; DDC, Dopa decarboxylase

The principal response in animals to external stress is the release of biogenic amines, with subsequent induction of hyperglycaemia [66]. Several biogenic amines, such as dopamine, norepinephrine, epinephrine, tyramine, octopamine, histamine and serotonin, have been reported in crustaceans [67]. The pathway for the biosynthesis of tyramine, octopamine, norepinephrine and epinephrine from tyrosine has been elucidated in bilaterians [68]. However, there are few reports of biogenic amines responding to WSSV infection in shrimp hemocytes. As shown in Fig. 6, norepinephrine (NE) and octopamine (OA) are both derived from the amino acid tyrosine via different pathways. In de novo pathway, OA biosynthesis from L-tyrosine is catalyzed by tyrosine decarboxylase (TDC) and then tyramine β-hydroxylase (TBH) [69]. Dopamine β-hydroxylase (DBH) is involved in the conversion of dopamine to norepinephrine in postganglionic sympathetic neurons [70]. Dopamine is transported into dopamine neurons by high-affinity dopamine transporter (HDT), which is critical in maintaining transmitter homeostasis [71] and may provide adequate substrates for the synthesis of NE. In the present study, these genes including TDC, TBH, DBH and HDT were up-regulated significantly after WSSV infection. The present data revealed three biogenic amines (NE, OA and TA) were likely to be induced by WSSV infection. Evidence shows that the biogenic amines play critical roles in mediating immune responses and metabolism via G protein-coupled receptor (GPCR) in target organs. In oyster hemocytes, NE binds to the receptors, resulting in a decline in cellular immune responses and an increase in mortality after a standard bacterial challenge [72]. In insect, OA induces mobilization of lipids and carbohydrates in target tissues, preparing for increased energy demand [69]. Furthermore, OA also affects the activity and characteristics of hemocytes, such as hemocytic phagocytosis and nodule formation [69, 73].Therefore, we suggested that the shrimp hemocytes perform rapid release of various biogenic amines upon WSSV infection, which might act on a variety of organs including itself via GPCR, and lead to a series of related responses in the entire body, including immune responses and metabolism.

#### Analysis of acetylcholine and its pathway in *L. vannamei*

**Fig. 7:**
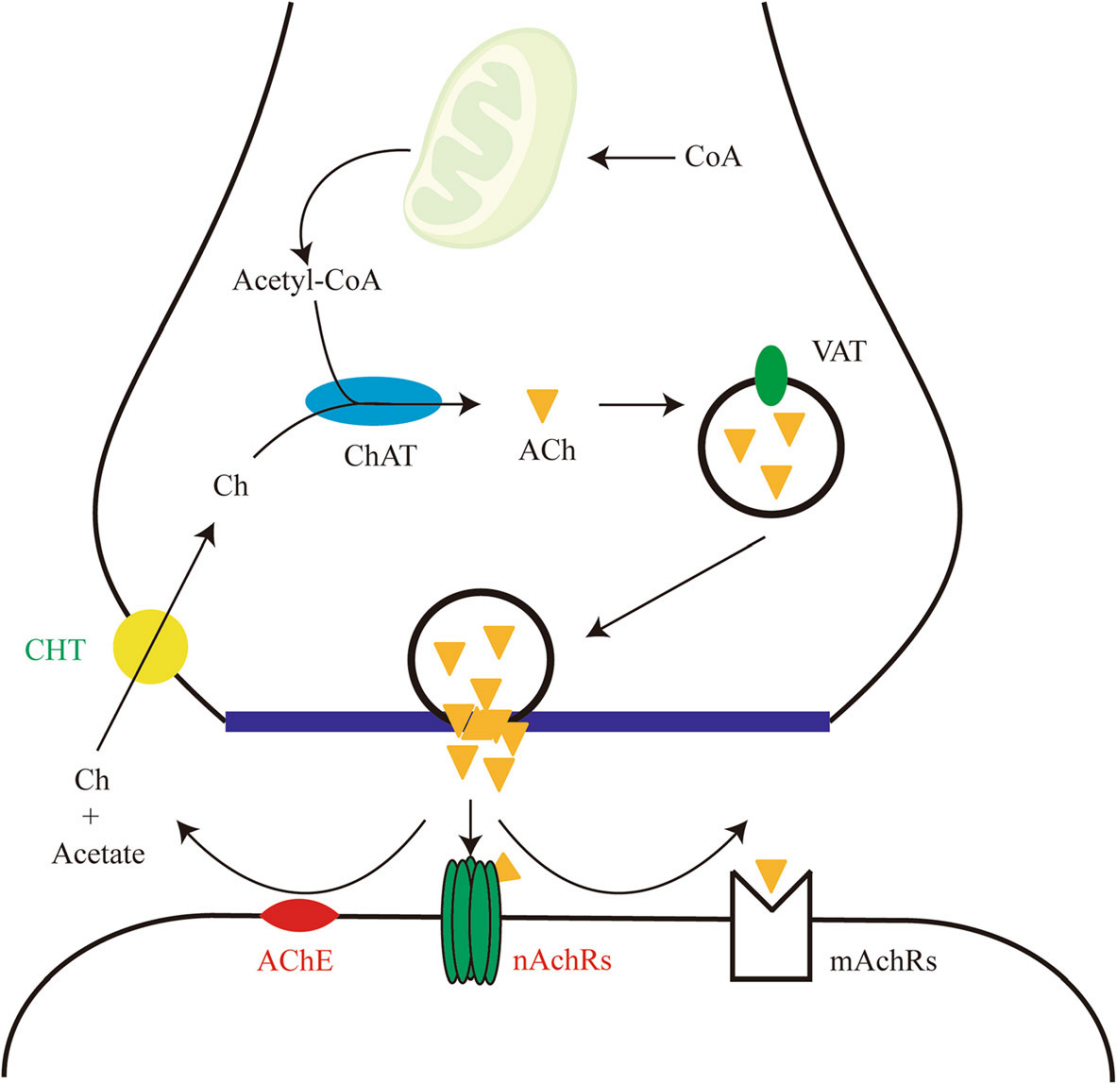
Schematic representation of cholinergic system. Red font denotes DUGs and green font denotes DDGs

Acetylcholine (ACh) is a component of the cholinergic system [74] and functions as an anti-inflammatory signal by binding to nicotinic acetylcholine receptors (nAChRs) on macrophages and inhibiting downstream NF-κB signaling [75]. Acetylcholinesterase (AChE) hydrolyses ACh into acetic acid and choline, which are re-absorbed from the extracellular space to presynaptic terminals by high-affinity choline transporter (CHT) [76]. Our data revealed that three AChE and three nAChRs subunits transcripts were significantly up-regulated in hemocytes upon WSSV infection (Fig. 7, Additional file 10). One of three AChE transcripts encodes a complete membrane protein and the other two encode different parts of a secreted protein, which are capable of reducing the acetylcholine level in the synaptic cleft and hemolymph. Besides, CHT was significantly down-regulated in hemocytes upon WSSV infection, which might block choline absorption and synthesis. We deduce that ACh levels in shrimp hemocytes are regulated during WSSV infection, which in turn affect the host’s immune response.

## Conclusions

In total, the transcriptional response of shrimp hemocytes to WSSV infection at early stage was studied by transcriptome analysis. A total of 3444 DEGs were identified in hemocytes after WSSV infection, and most of them are up-regulated. The immune-related DEGs consisted of pattern recognition, related to signal transduction, antimicrobial peptides, ubiquitin mediated proteolysis, related to phagocytosis and proteases/protease inhibitors. Among the DEGs, we found the genes related to the NEI system, including proteolytic processing of prohormones, amino acid neurotransmitter pathways, biogenic amine biosynthesis and acetylcholine signaling pathway, were apparently up-regulated. Therefore, we discussed their possible function during the early stage of WSSV infection. To our knowledge, it is a more comprehensive report on the composition of NEI system in shrimp hemocytes and the first time to report the role of NEI system in shrimp hemocytes upon WSSV infection. Consequently, our data enriches the knowledge about the immune function of hemocytes during pathogen infection and provides new perspective for developing disease-resistant technologies.

## Methods

### Experimental animals and WSSV challenge

The healthy WSSV-free shrimp *L. vannamei* (body weight: 9–10 g) used in the study were collected from laboratory culture tanks. The shrimp were fed thrice daily with artificial food pellets for 3 days before processing.

For in vivo WSSV challenge group, 15 individuals were randomly and equally divided into three parallel subgroups as biological replicates. Each shrimp was injected into 1000 copies of live WSSV particles suspended in 10 μl sterile phosphate-buffered saline (PBS) at the site between abdominal segments III and IV. In the control group, 15 individuals divided into three parallel subgroups were injected with the same volume of PBS.

### Sample preparation

At 6 hpi, approximately 500 μl hemolymph was collected from each shrimp using syringe containing an equal volume of shrimp anticoagulant solution (450 mM NaCl, 10 mM KCl, 10 mM EDTA, 10 mM Tris-HCl, pH 7.5). Samples of the hemolymph from five shrimps were mixed gently and centrifuged at 1000 g for 10 min at 4 °C. After centrifugation, pellets were present at the bottom of tube and stored in liquid nitrogen for total RNA isolation. The samples from WSSV challenge group and control group were designated as WHc and PHc, respectively.

Total RNA from frozen hemocytes was isolated with RNAiso Plus (TaKaRa, Japan) following the manufacturer’s instructions. The yield and purity of each RNA sample were evaluated using a NanoDrop™ 2000 spectrophotometer (Thermo Scientific, USA), and the integrity of all RNA samples was assessed by gel electrophoresis with 1.5% (*w*/*v*) agarose before sending for sequencing.

### Illumina sequencing

Next generation sequencing was conducted at Gene Denovo (Guangzhou, China) as per manufacturer’s protocol (Illumina, USA). Briefly, the eukaryotic mRNA was enriched by Oligo (dT) magnetic beads. All the mRNAs derived from fragmentation process were reverse-transcribed into the first strand cDNA using reverse transcriptase and random primers. The second-strand cDNA was synthesized using buffer, dNTPs, RNase H and DNA polymerase I. Then all products were purified using QiaQuick PCR extraction kit (Qiagen, Germany) and resolved with elution buffer for end reparation, adding of poly (A) and ligation with Illumina sequencing adapters. After the agarose gel electrophoresis, the suitable fragments were selected for the PCR amplification as templates. Ultimately, the libraries were sequenced using Illumina HiSeq™ 4000.

### Bioinformatics analysis

Raw image data obtained from the sequencing instrument was transformed to raw reads by base calling and stored in fastq format. To get high-quality clean reads, algorithms were run for removing empty reads, adaptor sequences and low-quality sequences. The clean reads of each group were then assembled into unigenes using RNA-Seq de novo assembly program Trinity [77], followed by TIGR Gene Indices clustering tools (TGICL) [78], with default parameters. Subsequently, blast alignment against four protein databases (Nr, Swiss-Prot, KEGG, KOG) was performed with a typical cut-off *E*-value of 10^− 5^ for annotation analysis, and the best alignments were used for further analysis. The gene abundances were calculated and normalized to reads per kilobase per million reads (RPKM). Genes were regarded as differentially expressed genes (DEGs) based on the RPKM value in PHc and WHc groups, followed by a multiple hypothesis testing: false discovery rate (FDR) < 0.05 and absolute value of log2 fold change (FC) > 1. DEGs were then subjected to enrichment analysis of GO functions and KEGG pathways by Blast2GO program [79] and KAAS (KEGG Automatic Annotation Server) [80], respectively.

For searching the molecular components of NEI network from all DEGs in shrimp hemocytes, the list of the annotated DEGs were manually scanned one by one according to previously existing knowledge. Selected cDNA sequences of DEGs were then converted to amino acids using Expasy translate tool (https://web.expasy.org/translate/). These deduced peptides were re-validated using blastp algorithm and their structural domains were predicted by SMART (http://smart.embl-heidelberg.de/). The structures of mature peptides were predicted using a well-established workflow [81].

### Validation of candidate genes by semi-quantitative PCR (SQ-PCR)

To verify the accuracy of RNA-seq data, SQ-PCR was performed using premix *Ex Taq* mix (Takara, Japan). A subset of DEGs involved in the response to WSSV infection were selected for validation and 18S rRNA gene was used as an internal standard. All primers were designed with PRIMER 5.00 (Premier Biosoft, USA) and the primers’ information was listed in Additional file 11. Pre-experiments were performed to quantify equal amounts of template and explore the appropriate number of amplification cycles. The amplified products of cDNAs from different samples were assessed by electrophoresis on 1.5% (*w*/*v*) agarose gel.

## Additional files

Additional file 1: The length distribution of predictive coding sequence (CDS). (TIF 90113 kb)

Additional file 2: Venn diagram of unigenes annotation from four public protein databases. (TIF 19258 kb)

Additional file 3: The data of all 3444 DEGs. (XLSX 481 kb)

Additional file 4: SQ-PCR verification of 15 DEGs in shrimp hemocytes. 18S rRNA gene was used as an internal standard. DDGs includes strongly chitin-binding protein-1 (SCBP-1) and protein CBR-CLEC-223 (223). Others are DUGs. (TIF 74027 kb)

Additional file 5: GO enrichment analysis of all 3444 DEGs. (XLSX 106 kb)

Additional file 6: GO term (level 2) distribution for the transcriptomes of *L. vannamei*. The x-axis indicates the name of GO subcategories. The y-axis represents the number of genes. Red displays up-regulated expression and green displays down-regulated expression as shown in the upper right corner of the picture. (TIF 14363 kb)

Additional file 7: KEGG pathway analysis of all 3444 DEGs. (XLSX 25 kb)

Additional file 8: The top 20 KEGG pathways enriched in shrimp hemocytes. “Rich factor” means that the ratio of the DEGs number to the number of all genes annotated in this pathway. The Rich factor is proportional to the degree of enrichment. (TIF 17740 kb)

Additional file 9: Detail information of 349 immune-related DEGs. (XLSX 48 kb)

Additional file 10: DEGs involved in NEI system. (XLSX 21 kb)

Additional file 11: Primers used for SQ-PCR validation. (DOCX 14 kb)

## Abbreviations

ACh: Acetylcholine
DEGs: Differentially expressed genes
EAAs: Excitatory amino acids
IAAs: Inhibitory amino acids
KAAS: KEGG Automatic Annotation Server
NE: Norepinephrine
NEI: Neuroendocrine-immune
OA: Octopamine
PCs: Prohormone convertases
PRRs: Pattern recognition receptors
RPKM: Reads per kilobase per million reads
SQ-PCR: Semi-quantitative PCR
WSSV: White spot syndrome virus
