# Supplementary figures and images for: Transcriptome analysis reveals the activation of neuroendocrine-immune system in shrimp hemocytes at the early stage of WSSV infection
Source: BMC Genomics. 2019 Mar 28;20:247. doi: 10.1186/s12864-019-5614-4 (PMC6437892; doi:10.1186/s12864-019-5614-4)

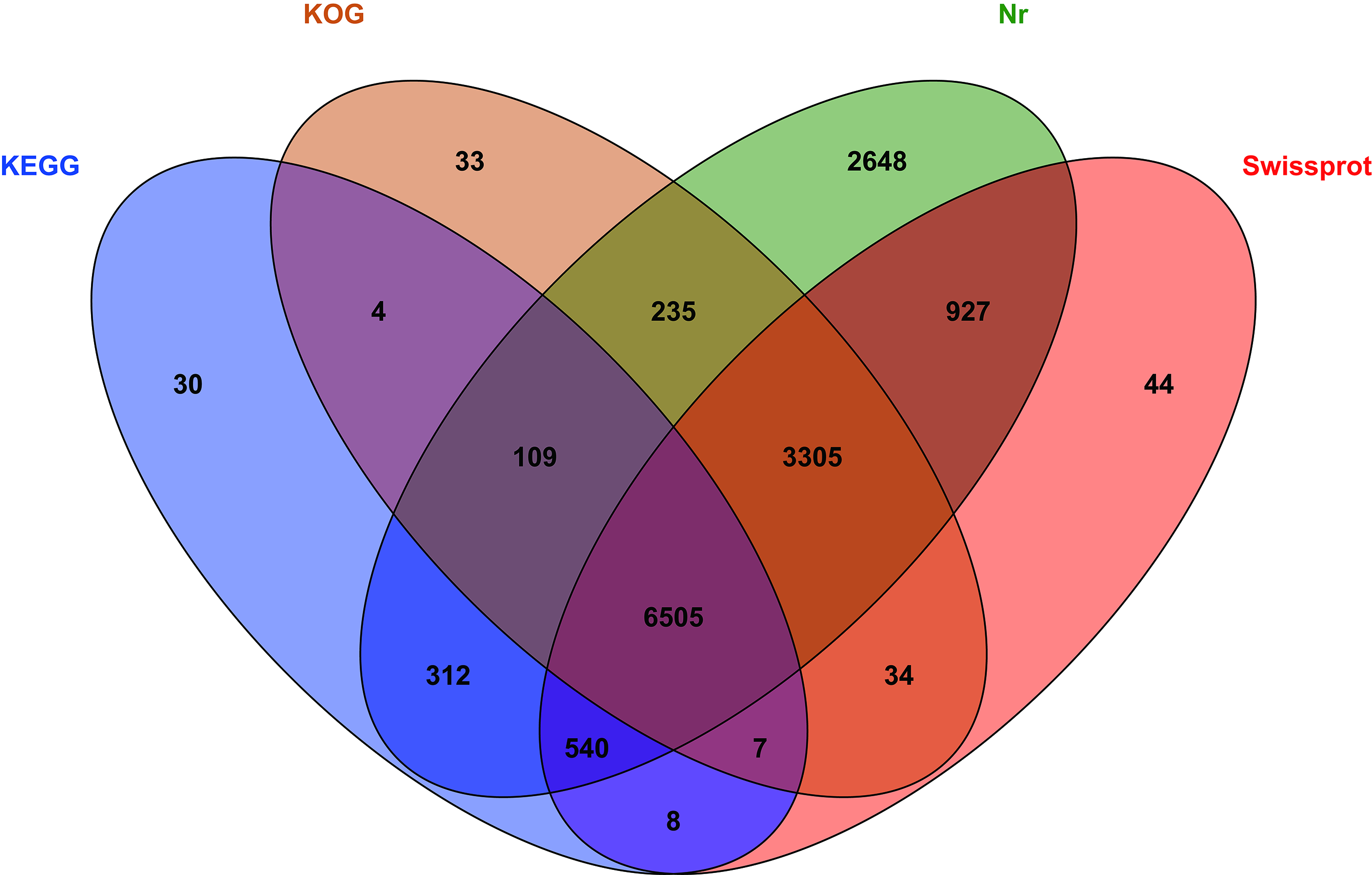

Supplement: Supplementary file 2 — Venn diagram of unigenes annotation from four public protein databases. (TIF 19258 kb) [file 12864_2019_5614_MOESM2_ESM.tif]

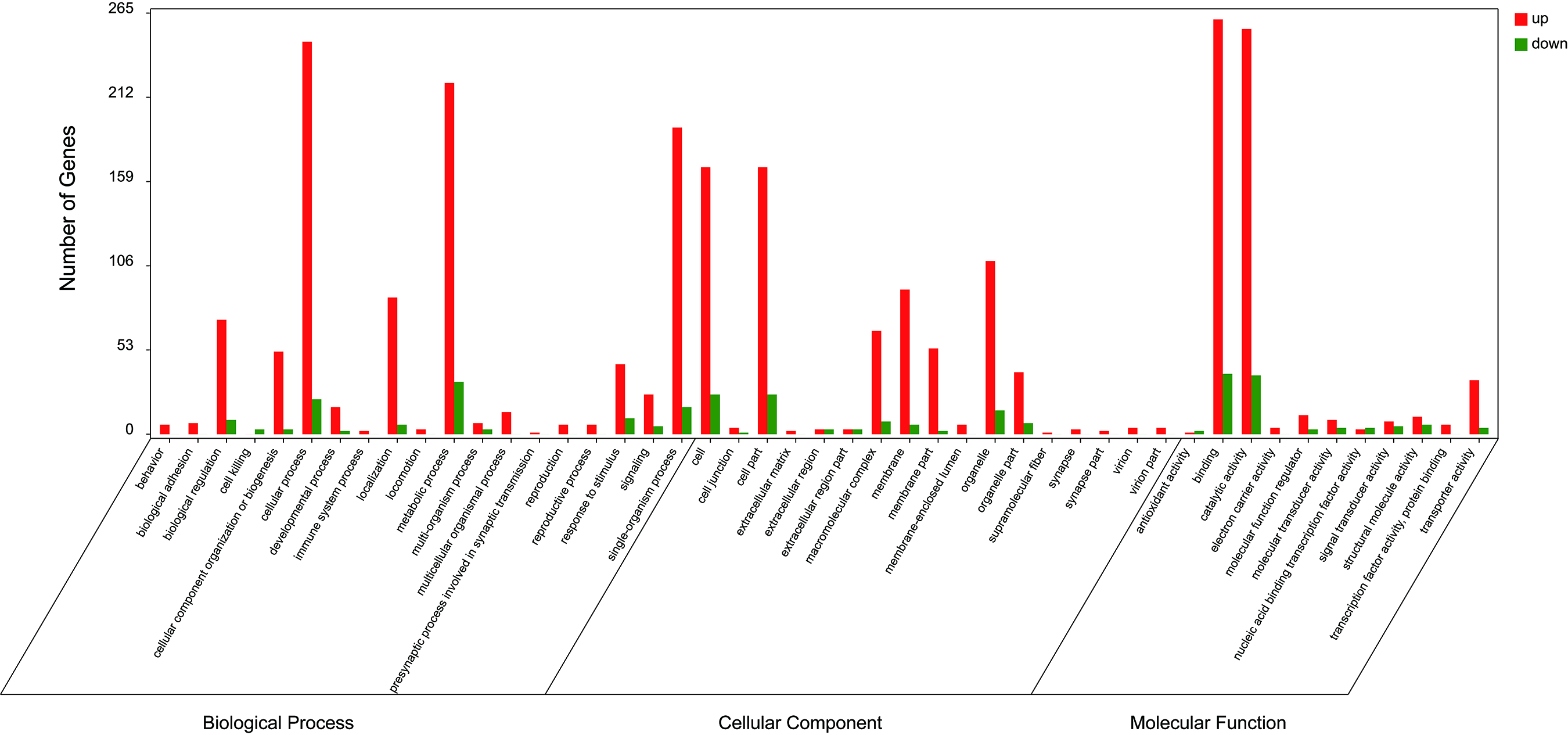

Supplement: Supplementary file 6 — GO term (level 2) distribution for the transcriptomes of L. vannamei. The x-axis indicates the name of GO subcategories. The y-axis represents the number of genes. Red displays up-regulated expression and green displays down-regulated expression as shown in the upper right corner of the picture. (TIF 14363 kb) [file 12864_2019_5614_MOESM6_ESM.tif]

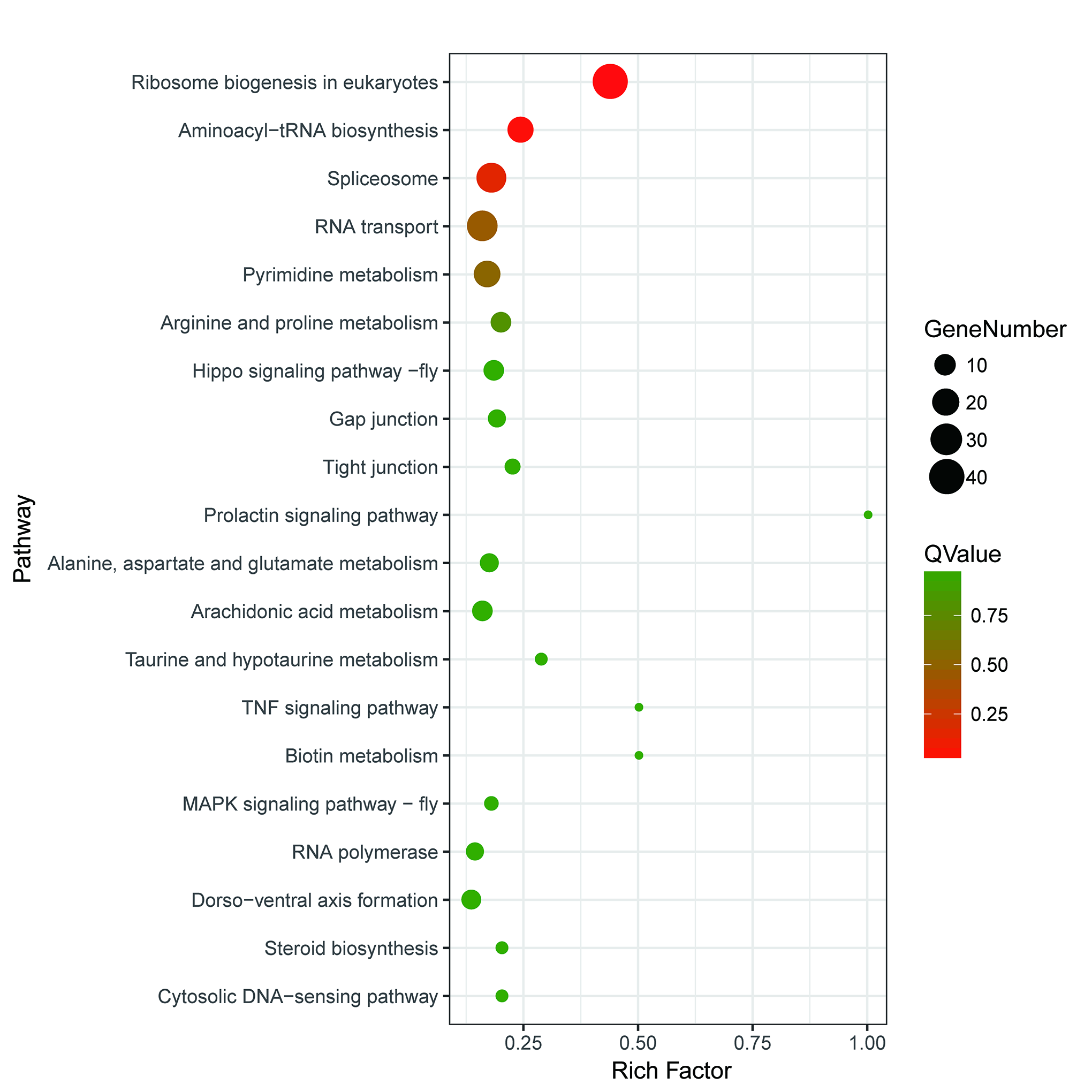

Supplement: Supplementary file 8 — The top 20 KEGG pathways enriched in shrimp hemocytes. “Rich factor” means that the ratio of the DEGs number to the number of all genes annotated in this pathway. The Rich factor is proportional to the degree of enrichment. (TIF 17740 kb) [file 12864_2019_5614_MOESM8_ESM.tif]
