# Supplementary material for: Transcriptome analysis reveals the activation of neuroendocrine-immune system in shrimp hemocytes at the early stage of WSSV infection
Source: BMC Genomics. 2019 Mar 28;20:247. doi: 10.1186/s12864-019-5614-4 (PMC6437892; doi:10.1186/s12864-019-5614-4)
Supplement: Supplementary file 11 — Primers used for SQ-PCR validation. (DOCX 14 kb) [file 12864_2019_5614_MOESM11_ESM.docx]

Table S1 Primers used for SQ-PCR validation.

| Gene name | Sequence (5' to 3') | Product size (bp) |
| --- | --- | --- |
| 18S | TATACGCTAGTGGAGCTGGAA | 147 |
|  | GGGGAGGTAGTGACGAAAAAT |  |
| AST-A | GTAGAGGGATTGAGGAGGGAA | 299 |
|  | GGGATACTATGGAATAAAACTAAAA |  |
| AST-B | AAACGGGCAACAGAGAATGA | 182 |
|  | TGTGGGAGGGAGGGTAAATC |  |
| Burs β | ATCAGTGTTACGGGGCAAGT | 223 |
|  | GACATAAATGACAAAGGGGTAAA |  |
| CCAP | GAAACACGATGCCAAAAAAC | 252 |
|  | CGACAAGCAGTGAGGAAGAA |  |
| CLDH | GAAAAAAAACACACAAACACATT | 163 |
|  | CTAAACGAAGCCATAACACAGA |  |
| NPF I | ACTTCTCGCACCTTCTCCTC | 170 |
|  | ACCATTTTCTCGGCACTTTTA |  |
| PC2 | TCAGCAACGGAGCCAAGGAC | 279 |
|  | GCACACCGAAGCCGAAGAGAT |  |
| 7B2 | GCTGACCCCTTATCACTGC | 206 |
|  | GTTGCTAAGAAGTTCCACCAGA |  |
| PAL2 | GAGATTGTGTGCTCTTCGG | 202 |
|  | TTGGTTCCTGGGTAGATGAT |  |
| TDC | AGGTTCGTTCCCTTCAGTCTG | 170 |
|  | CTGTGGTTCGTGATCCGCT |  |
| TBH | AGCATCAAGAAAGGGTTGAAG | 230 |
|  | CGTGGTAACGAGGACATAGTG |  |
| GABAT | CTGGATAATAAGAAACGCACAT | 168 |
|  | CTTGGAAGCCTTGAAAAACA |  |
| AChE1 | CGAGTTCTTATTGGCTTGGTT | 132 |
|  | GTTTTGGACCTGCTTCTCTG |  |
| SCBP-1 | TTGGCGATGAACTTGACCTC | 289 |
|  | CCACACCTGGCTACGAATACC |  |
| 223 | GCCAGTCAGTTTCGCAGTGT | 181 |
|  | GGCAAGTCTCGTCAATGTGTC |  |
